# Differential Spreading of Microsatellites in Holocentric Chromosomes of Chagas Disease Vectors: Genomic and Evolutionary Implications

**DOI:** 10.3390/insects14090772

**Published:** 2023-09-19

**Authors:** Francisco Panzera, Ángeles Cuadrado, Pablo Mora, Teresa Palomeque, Pedro Lorite, Sebastián Pita

**Affiliations:** 1 Evolutionary Genetic Section, Faculty of Science, University of the Republic, Iguá 4225, Montevideo 11400, Uruguay; fcopanzera@gmail.com; 2 Department of Biomedicine and Biotechnology, University of Alcalá (UAH), Alcalá de Henares, 28805 Madrid, Spain; angeles.cuadrado@uah.es; 3 Department of Experimental Biology, Genetics, University of Jaén, 23071 Jaén, Spain; pmora@ujaen.es (P.M.); tpalome@ujaen.es (T.P.)

**Keywords:** chromosome evolution, holocentric chromosomes, microsatellites, non-denaturing fluorescence in situ hybridization, repetitive DNA sequences, Triatominae

## Abstract

**Simple Summary:**

This study analyzed microsatellite distribution in the holocentric chromosomes of Triatominae, Chagas disease vectors. Using a non-denaturing FISH technique, 16 microsatellites were examined across 25 species from the Triatomini and Rhodniini tribes. Three hybridization patterns emerged: strong signals in specific regions, dispersed signals based on microsatellite abundance and the absence of signals in certain regions or chromosomes. Rhodniini had weak and scattered signals, indicating low microsatellite abundance, while Triatomini showed diverse and abundant patterns, suggesting their significance in genomes. Particularly, all Triatomini species exhibited a high abundance of GATA repeats in the Y chromosome, unlike Rhodniini. This suggests the ancestral trait is specific to Triatomini. The study provides insights into microsatellite composition and distribution in Triatominae genomes, shedding light on their evolutionary processes and relationships with other reduviid groups.

**Abstract:**

This study focused on analyzing the distribution of microsatellites in holocentric chromosomes of the Triatominae subfamily, insect vectors of Chagas disease. We employed a non-denaturing FISH technique to determine the chromosomal distribution of sixteen microsatellites across twenty-five triatomine species, involving five genera from the two principal tribes: Triatomini and Rhodniini. Three main hybridization patterns were identified: strong signals in specific chromosomal regions, dispersed signals dependent on microsatellite abundance and the absence of signals in certain chromosomal regions or entire chromosomes. Significant variations in hybridization patterns were observed between Rhodniini and Triatomini species. Rhodniini species displayed weak and scattered hybridization signals, indicating a low abundance of microsatellites in their genomes. In contrast, Triatomini species exhibited diverse and abundant hybridization patterns, suggesting that microsatellites are a significant repetitive component in their genomes. One particularly interesting finding was the high abundance of GATA repeats, and to a lesser extent AG repeats, in the Y chromosome of all analyzed Triatomini species. In contrast, the Y chromosome of Rhodniini species did not show enrichment in GATA and AG repeats. This suggests that the richness of GATA repeats on the Y chromosome likely represents an ancestral trait specific to the Triatomini tribe. Furthermore, this information can be used to elucidate the evolutionary relationships between Triatomini and other groups of reduviids, contributing to the understanding of the subfamily’s origin. Overall, this study provides a comprehensive understanding of the composition and distribution of microsatellites within Triatominae genomes, shedding light on their significance in the evolutionary processes of these species.

## 1. Introduction

Microsatellites, also known as short tandem repeats (STRs) or simple sequence repeats (SSRs), are tandem repetitions of 1–6 bp motifs that are present in both coding and non-coding regions of eukaryotic genomes [1]. These sequences undergo rapid expansions and contractions due to mechanisms such as replication slippage [2] and unequal crossing-over during meiosis [3,4], resulting in variation in the microsatellite length even among individuals of the same population. These characteristics make microsatellites valuable molecular markers for genetic mapping, allowing the analysis of genome organization, structure and evolution in a wide variety of organisms such as plants, fish and mammals [5,6].

Several search tools are available for mining microsatellite repeats in genomic libraries or in sequenced genome databases. In the last years, several reports about scanning genome sequences have provided important data for comparative analysis of microsatellite distribution and frequency in eukaryotic genomes, including those of insects [7,8,9]. While genomes may contain long microsatellite clusters (up to 500 nucleotides), these loci are usually no longer than 100 nucleotides. These short microsatellite arrays, despite their presence at many genomic locations, can only be detected in specific situations in the chromosomes by fluorescence in situ hybridization (FISH). As a consequence, they are cytologically invisible unless the density (loci/Mb) along the length of chromosomes or at specific genomic locations is significantly high [10]. However, in many species, certain microsatellite motifs are organized in long arrays consisting of several hundreds to thousands of tandem units, which can be either continuous or interrupted stretches extending over several kilobases. These long arrays of microsatellites, similar to other classes of tandemly highly repeated satellite sequences, are mainly localized in the heterochromatin and can be visualized using fluorescence in situ hybridization [11]. FISH techniques can be time-consuming due to the enzymatic pre-treatment of the cytological preparation, probe labeling and denaturing of probes and chromosomes. Non-denaturing FISH (ND-FISH) can be an alternative to conventional FISH techniques when oligonucleotides are used as probes [12]. The implementation of ND-FISH using single-sequence oligonucleotides as probes has been a breakthrough in determining the presence and distribution of microsatellites in both plants and animals, facilitating the knowledge of the chromosomal origin, organization, function and evolution of these repeats’ sequences [12,13,14].

In insects, bioinformatic analyses conducted on 136 species, including 12 hemipteran species, have revealed that microsatellites represent only a small fraction of the whole genome, ranging from 0.02% to 3.1% [8]. However, in two *Triatoma* species, *T. infestans* and *T. delpontei*, our genome analyses from low-coverage sequencing using RepeatExplorer2, RepeatMasker v.4.1.1 and RepeatProfiler software programs have identified two highly amplified microsatellites (GATA and CATA) [11,15]. The GATA repeats represented 14.21% of the *T. delpontei* genome and 3.20% and 6.24% of the *T. infestans* genomes, non-Andean and Andean lineages, respectively. A similar study in another triatomine species, *Rhodnius prolixus*, revealed that these microsatellites were not amplified in its genome [16].

Despite the large number of insect genomes explored by bioinformatics, very little is currently known about the chromosome location of microsatellites. The physical mapping of microsatellites is limited to a few species of Diptera [12,17], Orthoptera [10,18,19,20,21] and Hymenoptera [22,23,24,25].

The Triatominae subfamily (Hemiptera, Reduviidae) comprises over 150 species of blood-sucking insects commonly known as kissing bugs. Many of these species are vectors of Chagas disease [26], which is recognized as the most severe parasitic disease in Latin America. It is caused by the protozoan *Trypanosoma cruzi* and affects approximately six to seven million people worldwide [27]. Triatomines, like other hemipteran insects, possess chromosomes with diffuse or non-localized centromeres, known as holocentric chromosomes [28]. Triatomines exhibit high uniformity in their diploid chromosome numbers, ranging from 2n = 21 to 2n = 25 chromosomes in males [29]. The number of autosomes remains remarkably constant, with almost all of the 102 studied species having 20 autosomes (A), two species with 18A and one species with 22A [29]. In males, triatomines possess three sex chromosome systems: XY, X_1_X_2_Y and X_1_X_2_X_3_Y, the first one considered the ancestral system [30]. Despite the overall stability in chromosome number, triatomines exhibit significant karyotypic variation in terms of the quantity, distribution and base pair composition of heterochromatin, as evidenced by C-banding and fluorochrome staining techniques [29,31]. Additionally, there is considerable variability in the chromosomal location of the major ribosomal clusters among these insects [32].

To gain a deeper understanding of the microsatellite composition of heterochromatin and the sex chromosome evolution in the subfamily Triatominae (Hemiptera–Reduviidae), we mapped on the chromosomes of Triatominae representatives 16 microsatellite repeats. We used all possible classes of mononucleotide and trinucleotide repeats, as well as the dinucleotides AG and AC and the tetranucleotides GATA and GACA in the *Triatoma infestans* Andean group. According to the results obtained in *T. infestans*, among the sixteen microsatellite motifs tested, four microsatellite motifs (AG, AC, AAG and GATA) were the most suitable chromosomal markers to investigate their distribution in 24 triatomine species. These species belong to five genera and are included in two main tribes: Triatomini and Rhodniini. The studied species involved all known variations in autosomal numbers (2n = 18, 20, and 22) and male sex systems (XY, X_1_X_2_Y and X_1_X_2_X_3_Y) reported in Triatominae. Furthermore, these species also display variations in the amount and chromosomal location of autosomal heterochromatin. Through this approach, we anticipate obtaining a comprehensive overview of the composition and distribution of microsatellites in both autosomes and sex chromosomes. These data provided a broad and detailed understanding of the characteristics and dynamics of microsatellites in triatomine genomes, shedding light on their role in the evolutionary processes of Triatominae species.

## 2. Materials and Methods

### 2.1. Insects

Chromosome preparations for ND-FISH analyses were obtained from males of twenty-five triatomine species, including five genera of two principal tribes: Triatomini and Rhodniini. The geographic origins of analyzed triatomine species are provided in Supplementary Table S1. The classification of triatomine species into lineages and complexes followed the taxonomic subdivision proposed by Monteiro et al. [33], with subsequent modifications [34,35]. We studied species from the three main lineages of the Triatomini tribe, covering all variations in autosomal numbers (2n = 18, 20 and 22) and male sex chromosome systems (XY, X_1_X_2_Y and X_1_X_2_X_3_Y) reported within this group. Within the South American Triatomini lineage, we studied four species. For one species from this lineage, *T. infestans*, we analyzed the two chromosomal groups, Andean and non-Andean, which show striking differences in the amount and chromosome distribution of C-heterochromatin in both autosomes and sex chromosomes [36]. In the dispar lineage, we selected two species. In the North American lineage, we studied 17 species. For the Rhodniini tribe, we analyzed two species representing two of the three recognized lineages within this tribe (Supplementary Table S1).

### 2.2. Slide Preparation and C-Banding

Chromosome preparations were obtained from males. Testes were removed from adult insects alive and fixed in an ethanol:glacial acetic acid mixture (3:1). Fixed samples were stored at −20 °C. Squashes were prepared by placing a testis portion in a 50% acetic acid drop on the slide. The coverslips were removed after freezing in liquid nitrogen, and the slides were air dried and then stored at 4 °C. C-banding was performed according to Panzera et al. [36].

### 2.3. Probes and Labeling, Non-Denaturing (ND)—FISH

To identify the most suitable microsatellites as markers for chromosomal differentiation and explore their role as constituents of heterochromatin, we initially employed 16 microsatellite probes and performed ND-FISH on the chromosomes of the *T. infestans* Andean group. This species was chosen due to its abundance of constitutive heterochromatin and our comprehensive knowledge of its repeated sequence composition and variability, surpassing that of any other species [11,31,36,37,38]. The 16 microsatellite motifs were: all possible mononucleotides (A)_20_ and (C)_20_; the dinucleotide combinations (AG)_10_ and (AC)_10_; all trinucleotide combinations, (AAG)_5_, (AAC)_5_, (AGC)_5_, (ACT)_5_, (ATC)5_,_ (ACC)_5_, (AGG)_5_, (ACG)_5_, (AAT)_5_, (CCG)_5_; and two tetranucleotides, (GATA)_4_ and (GACA)_4_. Based on the mapping results obtained for *T. infestans*, the four oligonucleotides selected for the remaining 24 species were (AG)_10_, (AC)_10_, (AAG)_5_ and (GATA)_4_. All the probes were supplied with biotin-16-dUTP or digoxigenin-11-dUTP incorporated at 3′ and 5′ ends (Isogen Life Science, De Meern, The Netherlands).

ND-FISH analyses were carried out according to Cuadrado and Jouve [39]. It is worth mentioning that the protocol has been tested and optimized in several animal and plant species, showing that the obtained results were the same as those obtained by conventional FISH [12,13,39] and references therein. The slides were directly incubated at 24 °C in a humidity chamber for 2 h with 30 μL of hybridization mixture containing 2 pmol of the oligonucleotide probe in 2 × SSC. Afterward, the slides were washed in 4 × SSC/0.2% Tween20 with shaking for 10 min at RT. Digoxigenin and biotin were detected by incubating the slides with anti-digoxigenin-fluorescein (Roche Applied Science, Basel, Switzerland) and streptavidin-Cy3 (Sigma-Aldrich, Darmstadt, Germany), respectively, in 5% (*w*/*v*) bovine serum albumin for 1 h at 37 °C. The slides were then rinsed for 10 min in 4 × SSC/Tween20 at RT, stained with DAPI (4′,6-diamidino-2-phenylindole) and mounted with an antifade solution (Vectashield, Vector Labs, Burlingame, CA, USA).

### 2.4. Microscopy and Imaging

ND-FISH results were observed and photographed using a Zeiss Axiophot epifluorescence microscope equipped with an AxioCam MRm CCD camera (Zeiss, Jena, Germany). The images were compiled with AXIOVISIOM v4.8 software (Zeiss). The images were optimized for best contrast and brightness using Adobe Photoshop CS4 v.11.0. Hybridization pattern for each species was determined by analyzing the chromosomes of at least two individuals. After capturing ND-FISH signals on metaphase plates, some slides were washed and rehybridized with different microsatellite probes using the ND-FISH protocol described above.

## 3. Results

The application of microsatellite probes by ND-FISH showed three main hybridization patterns: (i) strong and discrete signals of variable size (blocks or dots) on specific chromosomal regions or entire chromosomes; (ii) scattered signals, with the intensity of hybridization depending on the microsatellite abundance on these regions; and (iii) some chromosomal regions or entire chromosomes did not exhibit any positive hybridization signals and remained free of labeling.

### 3.1. Abundance and Chromosomal Distribution of Microsatellites in Triatoma infestans Andean Group

**Figure 1:**
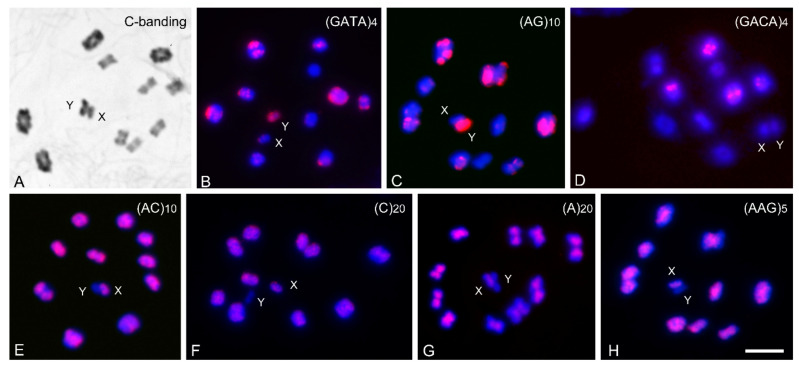
Male meiotic chromosomes (metaphase I) in *T. infestans* Andean group; 2n = 22 (10 bivalents and XY) with C-banding and mapping of several microsatellites. (**A**) C-banding. Almost all of the ten bivalents show C-heterochromatic bands in one or both chromosomal ends; only the smallest bivalent appears to lack heterochromatic regions. The Y chromosome appears entirely C-heterochromatic, while the X chromosome presents a small terminal C-region. (**B**–**H**) Hybridization signals (labeled in red) were obtained with different microsatellites on male meiotic chromosomes (labeled in blue). (**B**–**D**) Hybridization signals on several heterochromatic autosomal regions. In (**B**,**C**), the heterochromatic Y chromosome is labeled, while it is free of label in (**D**). (**E**–**H**) Hybridization signals on euchromatic regions. Scale bar = 10 μm.

In *T. infestans*, the Y chromosome is entirely heterochromatic, and the X chromosome has a small heterochromatic region [36]. The number of autosomes with C-blocks varies from four to twenty, with heterochromatic regions of different sizes at one or both chromosomal ends. This variation was observed among lineages (Andean and non-Andean), populations and individuals from the same population [36]. Figure 1A shows the C-banding pattern obtained in one of the Andean individuals used in this study, with heterochromatic blocks on nine of the ten bivalents; only the smallest bivalent seems to lack C-heterochromatic blocks. 

In *T. infestans*, three microsatellite probes, (GATA)_4_, (AG)_10_ and (GACA)_4_, generate strong hybridization signals on autosomal heterochromatic regions, showing motif-dependent hybridization patterns (Figure 1B, 1C and 1D, respectively). As commented above, the number of autosomes with heterochromatic blocks is variable. Anyway, the distribution and intensity of the (GATA)_4_ signals perfectly fit with the C-band distribution in this species (Figure 1A,B). The presence of GATA repeats in the heterochromatic regions of Andean *T. infestans* was previously shown using conventional FISH [11]. Two probes, (GATA)_4_ and (AG)_10_ also co-localized on the heterochromatic Y chromosome (Figure 1B,C). On the contrary, four motifs, (A)_20_, (C)_20_, (AC)_10_ and (AAG)_5_, showed dispersed hybridization signals along the majority of the length of all chromosomes except the heterochromatic Y chromosome. These repeats showed variation in hybridization intensity, indicating differences in their abundance and/or organization within the genome of this species (Figure 1E–H). For these four probes, hybridization signals appeared scattered over euchromatin regions of all autosomes and the X chromosome, covering almost the entire chromosomal length, except for the distal regions where heterochromatin was localized. It is noticeable that the hybridization patterns of the motifs (AG)_10_ and especially (GATA)_4_ are the reverse of the patterns of (A)_20_, (C)_20_, (AC)_10_ and (AAG)_5_, which correspond respectively to the distribution of heterochromatin and euchromatin in this species. For example, the heterochromatic Y chromosome exhibits hybridization signals with GATA and AG repeats (Figure 1B,C), while appearing free of labeling with GACA, AC, C, A and AAG repeats (Figure 1D–H). No hybridization signals were observed with the remaining analyzed trinucleotides.

### 3.2. Abundance and Chromosomal Distribution of GATA Repeats in Triatomine Species 

**Figure 2:**
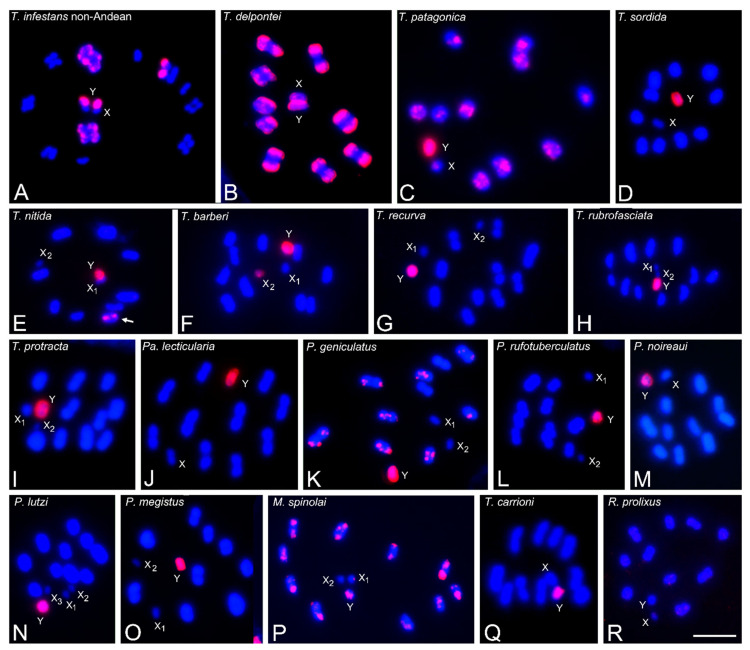
Microsatellite (GATA)_4_ hybridization signals (labeled in red) on male meiotic chromosomes (stained in blue) of several triatomine species. All Triatomini species exhibit strong hybridization signals on the heterochromatic Y chromosome. (**A**) *T. infestans* non-Andean group. Early anaphase I. Strong hybridization signals on heterochromatic regions of three autosomal pairs and the Y chromosome. (**B**) *T. delpontei*. MI. Strong signals are located at one chromosomal end of the ten bivalents. Both sex chromosomes (X and Y) appear labeled. (**C**) *T. patagonica*. Diakinesis. Heterochromatic regions of all bivalents and both sex chromosomes present GATA repeats. (**D**) *T. sordida*. MI. Autosomal heterochromatic blocks appear free of label. (**E**) *T. nitida*. MII. Dot signals are observed on one euchromatic half-bivalent (arrow). (**F**) *T. barberi*. MII. The smallest X_2_ and the Y chromosomes appear completely labeled. (**G**) *T. recurva*. MII. (**H**) *T. rubrofasciata*. MII. (**I**) *T. protracta*. MI. (**J**) *Pa. lecticularia*. MI. (**K**) *P. geniculatus*. MI. Eight of the ten bivalents exhibit hybridization dot signals at the heterochromatic chromosomal ends. (**L**) *P. rufotuberculatus*. MI. (**M**) *P. noireaui*. MI. (**N**) *P. lutzi*. MI. (**O**) *P. megistus*. MI. Only Y chromosome appears with hybridization signals. (**P**) *M. spinolai*. MII. GATA signals are observed on the heterochromatic terminal regions of the ten half-bivalents, on the Y chromosome (almost entirely), and on a dot region of the X_1_ chromosome. (**Q**) *T. carrioni*. MI. In several species (**D**–**F**,**H**–**J**,**L**,**M**), autosomal heterochromatic regions appear free of label. (**R**) *R. prolixus*. MI. No strong or accumulated signals were observed. Abbreviations: MI = first meiotic metaphase; MII = second meiotic metaphase. Scale bar = 10 μm.

The heterochromatic Y chromosome of all analyzed Triatomini species (23 species) exhibits strong hybridization signals with the (GATA)_4_ probe, similar to those shown in the *T. infestans* Andean group (Figure 1B) (Table 1). In addition, and similar to the *T. infestans* Andean group (Figure 1D), some species of *Triatoma*, *Panstrongylus* and *Mepraia* showed hybridization with (GATA)_4_ on C-heterochromatic regions of several autosomes. The number and size of the hybridization signals were variable according to the distribution of the C-heterochromatin (C-bands) in the respective species (Table 1) (Figure 2A–C,K,P). The presence of GATA repeats in the heterochromatin was previously described in Andean and non-Andean *T. infestans*, as well as in *T. delpontei*, using conventional FISH [11,15]. Interestingly, in other Triatomini species, the heterochromatic autosomal regions appear free of the GATA label, such as *T. sordida* (Figure 2D), all North American *Triatoma* (Figure 2E,F,H,I), *Paratriatoma lecticularia* (Figure 2J) and several *Panstrongylus* species (*P. chinai*, *P. rufotuberculatus*, *P. noireaui*, Figure 2L,M) (Table 1). However, *T. nitida* presents a euchromatic bivalent with strong GATA signals (Figure 2E, arrow). The remaining eight bivalents, including the two heterochromatic ones, do not have any GATA signals. In most species, the X chromosomes appear without hybridization signals, excepting some species with a GATA dot, such as the *T. infestans* Andean group (Figure 1B), *T. delpontei* (Figure 2B), *T. patagonica* (Figure 2C), and *M. spinolai* (Figure 2P). Nevertheless, in *T. barberi* (Figure 2F), the hybridization signals cover the entire length of the X_2_ chromosome. In these species, the X chromosomes also have a heterochromatic region revealed by C-banding (Table 1). No (GATA)_4_ signals were observed on any chromosomes (autosomes or sex chromosomes) of the two analyzed *Rhodnius* species (Figure 2R).

### 3.3. Abundance and Chromosomal Distribution of AG Repeats in Triatomine Species 

**Figure 3:**
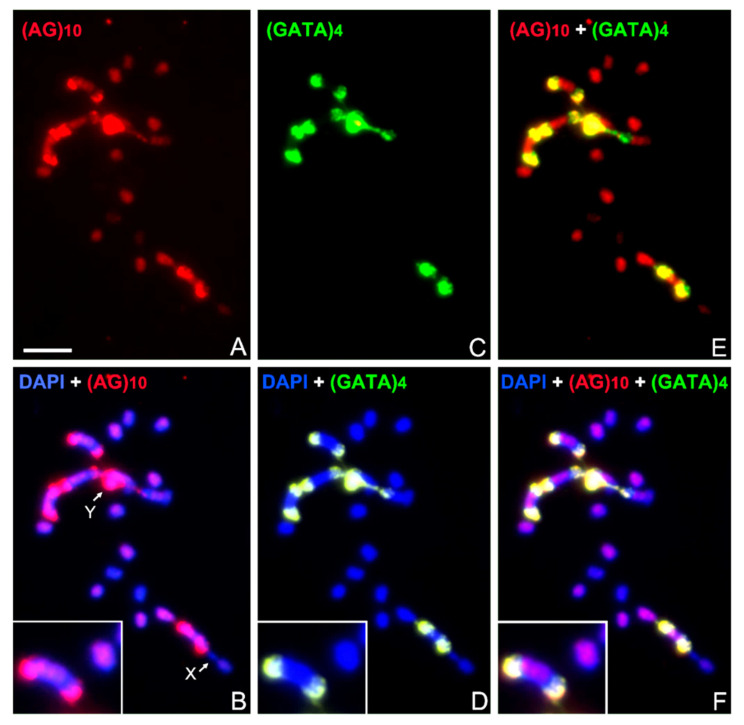
Hybridization on male mitotic chromosomes of *T. infestans* (non-Andean group) with (AG)_10_ (**A**,**B**) and (GATA)_4_ probes (labeled in red and green respectively). (**C**,**D**) and merged images (**E**,**F**). Both probes generate strong hybridization signals located on the heterochromatic regions of four autosomes and the Y chromosome, but dispersed AG repeats were also observed in most euchromatic autosomal regions (**A**,**B**,**E**,**F**) (more details in bottom boxes in (**B**,**D**,**F**)). Some euchromatic regions did not show signals with any of the two probes (**F**). Chromosomes stained with DAPI (blue).

This microsatellite generates three main hybridization patterns: (i) accumulated signals at heterochromatic regions of autosomes and sex chromosomes, (ii) dispersed signals on euchromatic regions and (iii) heterochromatic and euchromatic regions without hybridization signals (free of label). These patterns can be present in the chromosomes of the same species, as in *T. infestans* (Figure 3). In this species, AG repeats generate strong hybridization signals on heterochromatic regions of four autosomes and the Y chromosome (Figure 3A,B), similar to those observed with GATA repeats (Figure 3C,D). In addition, dispersed AG repeats are also observed in most euchromatic autosomal regions, while other euchromatic regions and the X chromosome did not show a label (Figure 3E,F).

**Figure 4:**
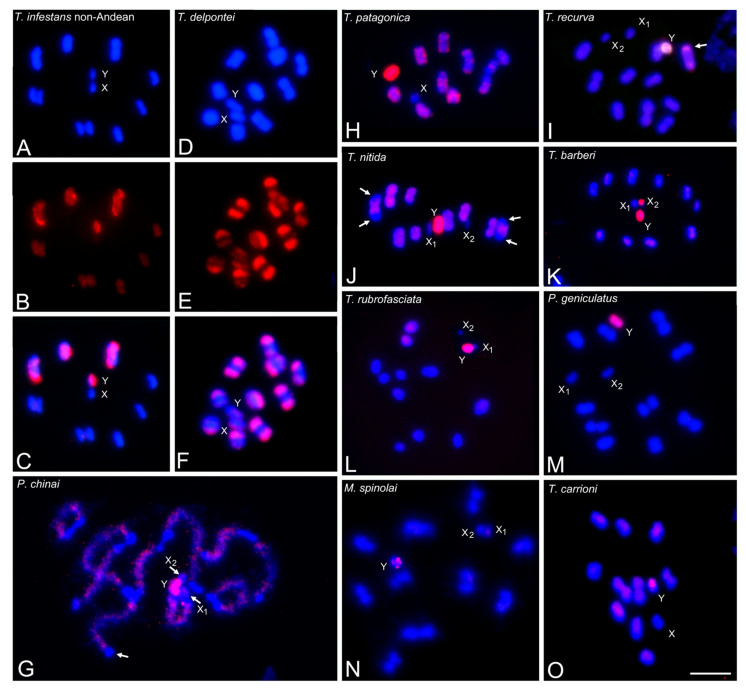
Microsatellite (AG)_10_ hybridization signals (labeled in red) on male meiotic chromosomes of several Triatomini species (stained in blue). All Triatomini species exhibit strong hybridization signals on the heterochromatic Y chromosome. (**A**–**C**) *T. infestans* non-Andean group. MI. (**A**) DAPI staining. (**B**) AG probe. (**C**) Merged A–B. Strong hybridization signals on heterochromatic regions of three bivalents and the Y chromosome. (**D**–**F**) *T. delpontei*. MI. (**D**) DAPI staining. (**E**) AG probe. (**F**) Merged A–B. Ten bivalents with accumulated signals. The sex chromosomes show less intense hybridization signals. (**G**) *P. chinai*. Pachytene. Autosomal heterochromatic regions (in blue) free of label, while euchromatic regions show scattered signals (in red). (**H**) *T. patagonica*. MI. Heterochromatic regions of the ten bivalents exhibit hybridization signals. (**I**) *T. recurva*. MI. Dot signals in one bivalent (arrow). (**J**) *T. nitida*. MI. The heterochromatic regions of two bivalents do not present hybridization signals (arrows). (**K**) *T. barberi*. MII. Besides the Y chromosome, the smallest X_2_ chromosome appears almost entirely labeled. (**L**) *T. rubrofasciata*. MI. (**M**) *P. geniculatus*. MI. (**N**) *M. spinolai*. Diakinesis. (**O**) *T. carrioni*. MI. In the last seven species, the autosomal heterochromatic regions appear free of label. Abbreviations: MI = first meiotic metaphase; MII = second meiotic metaphase. Scale bar = 10 μm.

In the other Triatomini species, the accumulated pattern of AG is also very similar to that observed with GATA repeats (Figure 4). The heterochromatic Y chromosome is also intensely labeled with the AG probes, indicating the high number of these repeats. In autosomal chromosomes, in some species, such as *T. infestans* (both groups) (Figure 3A and Figure 4C), *T. delpontei* (Figure 4F) and *T. patagonica* (Figure 4H), AG repeats colocalize in the same heterochromatic blocks with GATA repeats. However, in *T. sordida* and all North American *Triatoma*, *Panstrongylus*, *Paratriatoma* and *Mepraia* species (Figure 4G,I–O), the heterochromatic autosomal regions appear free of labeling with the (AG)_10_ probe, while the euchromatic regions show dispersed and weak signals. For example, autosomal heterochromatic regions in the ten bivalents of *P. chinai* (Figure 4G) and *T. nitida* (Figure 4J, two bivalents pointed by arrows) do not show AG repeats. Two species, *T. sanguisuga* and *T. recurva* (arrow, Figure 4I) show a strong dot signal in only one bivalent. The X chromosomes appear without hybridization signals in all species except for *T. barberi*, where the smallest X_2_ chromosome is almost entirely labeled (Figure 4K). No (AG)_10_ signals were observed in any chromosome of the two *Rhodnius* species analyzed.

### 3.4. Abundance and Chromosomal Distribution of AC Repeats in Triatomine Species

**Figure 5:**
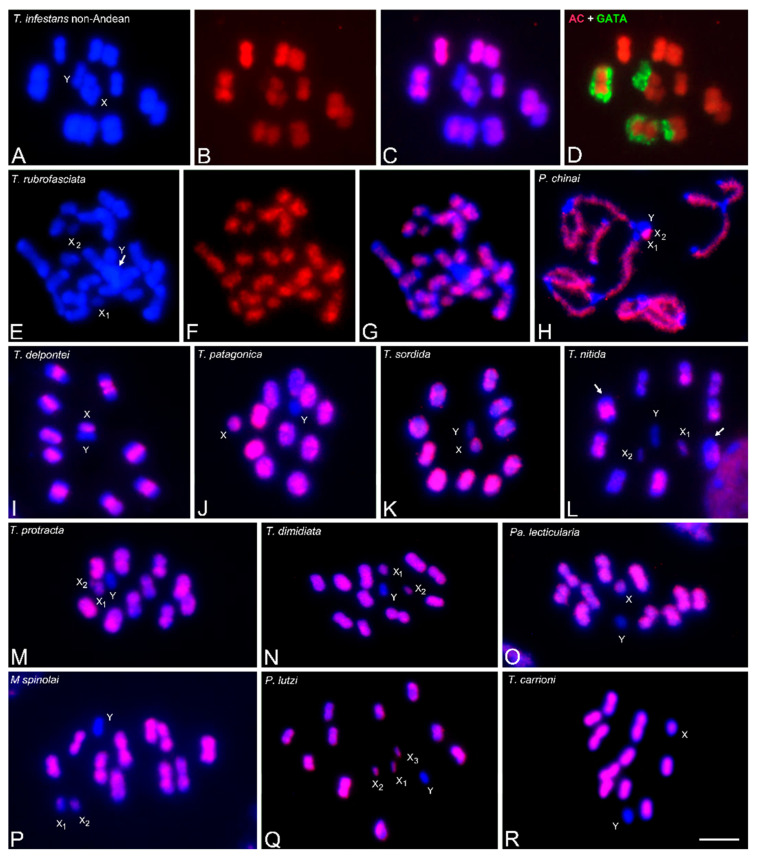
Microsatellite (AC)_10_ hybridization signals (labeled in red) on male chromosomes (stained in blue) of several Triatomini species. Hybridization signals appear scattered over euchromatin regions of autosomes and the X chromosomes. Heterochromatic regions of autosomes and sex chromosomes (X and Y) do not present hybridization signals. (**A**–**D**) *T. infestans* non-Andean group. MI. (**A**) DAPI staining shows 10 bivalents plus XY chromosomes. (**B**) (AC)_10_ hybridization signals over euchromatic regions of all bivalents and X chromosome. (**C**) Merged figures of A and B. (**D**) Double hybridization with AC + GATA demonstrating that the heterochromatic regions of three bivalents and the Y chromosome do not have AC signals (GATA regions labeled in green). (**E**–**G**) *T. rubrofasciata*. Spermatogonial prometaphase. (**E**) DAPI staining shows heterochromatic terminal regions in all autosomes and the Y chromosome. (**F**) (AC)_10_ hybridization signals over euchromatic regions of all autosomes and the X chromosomes. Arrow indicates the Y chromosome is free of label. (**G**) Merged figure of (**E**,**F**). (**H**) *P. chinai*. Pachytene. Euchromatin of autosomes with AC repeats while terminal heterochromatic autosomal regions of all bivalents and the Y chromosome lack AC signals. (**I**) *T. delpontei*. MI. (**J**) *T. patagonica*. MI. (**K**) *T. sordida*. MI. (**L**) *T. nitida*. Heterochromatic regions of two bivalents (arrows) without hybridization signals. (**M**) *T. protracta*. MI. (**N**) *T. dimidiata*. MI. Euchromatin of all autosomes and the two X chromosomes appear homogeneously labeled. (**O**) *Pa. lecticularia*. MI. (**P**) *M. spinolai*. MI. Small terminal heterochromatic regions of all bivalents and the Y chromosome appear without label. (**Q**) *P. lutzi*. MI. All autosomes and the three X chromosomes show hybridization signals, but the heterochromatic Y chromosome lacks them. (**R**) *T. carrioni*. MI. Similar to *P. lutzi*. Abbreviations: MI = first meiotic metaphase; MII = second meiotic metaphase. Scale bar = 10 μm.

In all analyzed Triatomini species, AC repeats were found dispersed over most of the euchromatin, both on autosomes and X chromosomes, with different levels of intensity depending on the species. The heterochromatic Y chromosome always appears free of the AC label in all Triatomini species. Additionally, AC repeats were absent on the heterochromatic regions of autosomes and X chromosomes (Figure 5D–P). The richness of AC repeats over most of the length of the autosomes could be observed in species lacking autosomal heterochromatin, such as *P. lutzi* and *T. carrioni* (Figure 5Q and Figure 5R, respectively). Figure 5E–G show the same spermatogonial metaphase of *T. rubrofasciata*, wherein the terminal C-heterochromatic regions of all autosomes and Y chromosomes (arrow) appear without AC signals, while the euchromatic regions are labeled. Notably, in some species, such as *T. delpontei*, the (AC)_10_ pattern (Figure 5I) showed an exact inverse pattern compared to the GATA pattern (Figure 2B). Finally, no signals with (AC)_10_ were observed in the analyzed *Rhodnius* species despite increasing the CCD camera exposure time.

### 3.5. Abundance and Chromosomal Distribution of AAG Repeats in Triatomine Species

**Figure 6:**
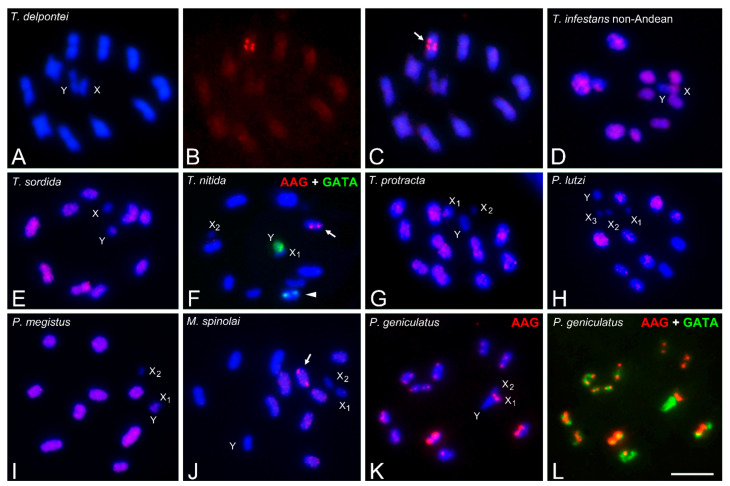
Microsatellite (AAG)_5_ hybridization signals (labeled in red) on male meiotic chromosomes of several Triatomini species (stained in blue). In all species, the euchromatic regions of the autosomes and sex chromosomes show dispersed and weak hybridization signals, while the Y chromosome and the autosomal heterochromatic regions remain unlabeled. Additionally, some species presented small regions of hybridization. (**A**–**C**) *T. delpontei*. MI. (**A**) DAPI staining. (**B**) AAG repeats. (**C**) Merged figure of (**A**,**B**). Dot AAG signals were observed on one single bivalent (arrow). (**D**) *T. infestans* non-Andean group. MI. (**E**) *T. sordida*. MI. Similar to *T. infestans*, only dispersed signals were observed over all euchromatic regions. (**F**) *T. nitida*. MII. Double hybridization, AAG + GATA. One half-bivalent presents AAG dots (arrow), distinct from the one observed with GATA repeats (arrowhead). (**G**) *T. protracta*. MI. (**H**) *P. lutzi*. MI. All chromosomes showed weak hybridization signals. (**I**) *P. megistus*. MI. MII. (**J**) *M. spinolai*. MII. One half-bivalent exhibits dot signals (arrow). (**K**,**L**) *P. geniculatus*. MII. (**K**) AAG pattern. (**L**) Double hybridization AAG + GATA. The euchromatin regions of all half-bivalents and both X chromosomes present small AAG signals, while the Y chromosomes remain unlabeled. The reverse pattern is observed with GATA repeats as a probe. GATA regions labeled in green. Abbreviations: MI = first meiotic metaphase; MII = second meiotic metaphase. Scale bar = 10 μm.

All Triatomini species exhibit (AAG)_5_ hybridization patterns that are similar to those observed with (AC)_10_ but less intense, i.e., dispersed signals over all euchromatic regions, while heterochromatic regions of autosomes and sex chromosomes appear free of label. All species have a heterochromatic Y chromosome without an AAG label. Only four species, not evolutionarily related, showed strong hybridization signals (Table 1). *Triatoma delpontei* (Figure 6B,C), *T. nitida* (Figure 6F) and *M. spinolai* (Figure 6J) show a dot region on one autosome pair. In *T. nitida* (Figure 6F, double hybridization AAG + GATA), the half-bivalent with (AAG)_5_ region (pointed by the arrow) differs from the half-bivalent carrying GATA repeats (arrowhead). In *P. geniculatus* (Figure 6K), the euchromatic regions of all autosomes and both X chromosomes presented terminal AAG regions, while the Y chromosome was free of label. The double hybridization of AAG + GATA (Figure 6L) clearly shows the reverse distribution of both microsatellites, with euchromatin labeled with AAG and heterochromatin labeled with GATA.

## 4. Discussion

This study represents the first analysis of the distribution and abundance of microsatellites in insect genomes with holocentric chromosomes. We explore the chromosomal location of 16 microsatellites in Andean *T. infestans*. According to the results obtained in this species, four of them were selected and mapped in a total of twenty-five triatomine species from five genera belonging to the two major tribes within the Triatominae subfamily: Rhodniini and Triatomini. The analyzed species encompassed the full range of numerical variations reported in this subfamily, including 18, 20 and 22 autosomes, as well as the three found male sex mechanisms (XY, X_1_X_2_Y and X_1_X_2_X_3_Y) [25].

The analysis of the chromosomal distribution of microsatellites yielded two main findings. First, significant differences in hybridization patterns were observed among species belonging to the Rhodniini and Triatomini tribes. In Rhodniini species, no discrete hybridization signals were observed in any chromosomal region. The hybridization signals appeared very scattered and weak, suggesting a low abundance of microsatellites in the genomes of Rhodniini species. These findings are consistent with previous bioinformatic analyses, which suggest that microsatellites constitute less than 1% of the genome of *R. prolixus* [8,16,43]. RepeatExplorer analysis also revealed the absence of amplification of these microsatellites in *R. prolixus* [16]. In contrast, the Triatomini species displayed diverse and abundant hybridization patterns, indicating that microsatellites constitute a significant component of repetitive DNA in their genomes. Previous studies using RepeatExplorer identified specific microsatellites, such as GATA and CATA, which accounted for more than 14.3% of the *T. delpontei* genome and between 3.9% and 8.8% of the *T. infestans* genomes (non-Andean and Andean lineages, respectively) [11,15]. In conclusion, species from the Rhodniini and Triatomini tribes display remarkable differences in the quantity and distribution of microsatellites.

The second interesting finding is that in all analyzed Triatomini species, the heterochromatic Y chromosome exhibits a high abundance of GATA repeats and, to a lesser extent, AG repeats. The richness of GATA repeats on the Y chromosome is likely an ancestral trait of the Triatomini tribe. In contrast, in the Rhodniini tribe, the Y chromosome does not show enrichment of GATA and AG repeats. These results are in line with previous GISH (genomic in situ hybridization) studies that employed five genomic probes on fifteen triatomine species from seven genera. These studies demonstrated that the heterochromatic Y chromosome in Triatomini is significantly enriched with highly repetitive sequences, while the Y chromosome in Rhodniini lacks repeated regions [37,38]. Furthermore, data obtained from sex chromosome painting using microdissection of the X chromosome also indicate a clear differentiation in the repeat sequence composition of the sex chromosomes between the Triatomini and Rhodniini tribes [44].

### 4.1. Microsatellite Patterns

Due to the hybridization patterns obtained in *T. infestans*, four microsatellites were selected to use as hybridization probes in other triatomine species: GATA, AG, AC and AAG. The GATA microsatellite is mostly restricted to heterochromatic regions, both on autosomes and sex chromosomes (Figure 2). Only one species, *T. nitida*, showed GATA signals outside of the heterochromatic regions (Figure 2E, arrows). In other insect species, GATA motifs were reported only in euchromatic and interstitial regions, such as in crickets [19] or grasshoppers [10,18]. The GATA microsatellite was present in the heterochromatic Y chromosome of all analyzed Triatomini species. In certain species, including Andean *T. infestans*, *T. delpontei* and *T. patagonica*, the X chromosome also exhibits heterochromatic regions enriched with GATA repeats (depicted in Figure 1 and Figure 2, and detailed in Table 1). In the autosomal chromosomes, some species have their heterochromatic regions enriched with GATA repeats (*T. infestans*, *T. delpontei*, *T. patagonica*, *T. boliviana*, *T. carrioni*, *P. geniculatus* and *M. spinolai*), while in other species, the heterochromatic regions lack this microsatellite (*T. sordida*, *T. barberi*, *T. dimidiata*, *T. nitida*, *T. rubrofasciata*, *T. sanguisuga*, *Pa. lecticularia*, *P. rufotuberculatus* and *P. noireaui*) (Table 1). Autosomal GATA repeats appear to be a derived trait, as they were observed in Triatomini species belonging to evolutionarily distant groups.

The AG microsatellite was also present in the Y chromosome of all Triatomini species, although in lower abundance compared to GATA repeats (Figure 3 and Figure 4). In all species, the X chromosomes showed no AG hybridization signals, except for *T. barberi* (Figure 4K), where the X_2_ chromosome exhibited strong labeling. This could indicate a possible origin of the X_2_ chromosome from the Y chromosome and not from a fission of the X_1_ chromosome, or at least the transference of repeat sequences from the Y chromosome to the X_2_ chromosome. In autosomal chromosomes, AG repeats are always dispersed on the euchromatin of all Triatomini species. However, some species displayed strong hybridization signals in the heterochromatic regions, such as *T. infestans* (Figure 3A and Figure 4C), *T. delpontei* (Figure 4F) and *T. patagonica* (Figure 4H). Other species did not show AG repeats in their autosomal heterochromatic regions, for example, *P. chinai* (Figure 4G). Interestingly, discrete AG signals were observed in one bivalent of *T. sanguisuga* and *T. recurva* (Figure 4I). The heterochromatic location of AG repeats in Triatomini is exceptional among insects since the common location of this repeat is the euchromatic regions, as observed in grasshoppers [10,18,20], crickets [19], ants [23], stingless bees [24,25,45], *Drosophila* [12] and social wasps [46].

The microsatellites AC and AAG were found scattered throughout the euchromatin, both on autosomes and X chromosomes, with varying intensity among species (Figure 5 and Figure 6). The heterochromatic Y chromosome and the autosomal heterochromatic regions consistently displayed no labeling with these microsatellites. With AAG motifs, several species presented discrete signals on specific autosomes (Figure 6C,F,J,K). In grasshopper species, these microsatellites were also dispersed across most euchromatic regions, with discrete signals often co-located with H3 histone genes [10].

### 4.2. Evolutionary Implications

In Triatomini species, the strongest hybridization signals with microsatellite probes occur in heterochromatic regions, while they are rarely found in euchromatic regions. However, several species have shown autosomal heterochromatic regions that do not contain the applied microsatellites. This suggests that the heterochromatic regions in triatomines are composed of a diverse variety of repetitive sequences, some of which may not include short repeats that typically characterize microsatellites. Long repeats of satellite DNA sequences have been described in *T. infestans* and *T. delpontei*, located in both the heterochromatin and autosomal euchromatin [11,15].

The presence of shared microsatellites among autosomes and sex chromosomes, such as GATA or AG motifs, observed in several evolutionarily distant species, suggests independent processes of dispersion. The transference of these repetitive sequences from the Y chromosome to the autosomes and/or to the X chromosome could be triggered by the heterologous associations between autosomes and sex chromosomes during the first meiotic prophase observed in several triatomine species, similar to what has been described for ribosomal clusters [32].

In all Triatomini species, hybridization signals on euchromatin appear homogeneously dispersed with AC and AAG motifs (Figure 5 and Figure 6). Only four not related triatomine species (*T. delpontei*, *T. nitida*, *M. spinolai* and *P. geniculatus*) exhibit discrete and strong, but small, hybridization signals using the AAG repeat as a probe (Figure 6). These regions are most likely the result of independent amplification processes of AAG repeats during the genomic evolution of these species.

In summary, the distribution of the applied microsatellites provides valuable insights into the evolution of autosomes and sex chromosomes in Triatominae. The extensive variation detected with microsatellites, along with that described for heterochromatic regions and the localization of ribosomal genes, demonstrates that the karyotypes of Triatominae undergo extensive chromosomal rearrangements, with the particularity of not affecting the number of chromosomes. The significant differentiation in microsatellite hybridization patterns and the distinct sequence composition of the Y chromosome among Rhodniini and Triatomini species raise questions about the accepted monophyletic origin of the Triatominae subfamily [33,47]. The abundance of GATA repeats on the Y chromosome in all analyzed Triatomini species likely represents an ancestral trait of this tribe that is not shared with Rhodniini. Therefore, it can be used as a comparative genomic marker with the putative predatory ancestors of triatomines such as the Zelurus clade and the *Opisthacidius* genus, a genus closely related to Rhodniini [47,48,49]. Preliminary analyses on the reduviid *Zelurus femoralis* revealed enrichment of GATA and AG repeats in its Y chromosome, like in the Triatomini tribe species. Further investigations in other species of the Zelurus clade and the *Opisthacidius* provide additional insights to resolve the controversial phylogeny of the Triatominae subfamily in relation to their closest predatory relatives.

## Figures and Tables

**Table 1:** Microsatellite patterns with (GATA)_4_, (AG)_10_, (AC)_10_ and (AAG)_5_ probes in 25 triatomine species here studied, including their diploid chromosome number (2n) in males and chromosome location of autosomal C-heterochromatin. Polymorphism for the C-banding pattern is indicated for the autosomes (A) as the number of bivalents (II) with heterochromatin, and the location of the blocks. Equally, the number of bivalents with hybridization signals is indicated for each microsatellite. Also, the location was specified to depict whether the signals are in the euchromatic region (Eu) or in the heterochromatic blocks (H). For the sexual chromosomes, “yes” indicates that hybridization signals cover the entire chromosome, and “block” indicates the presence of a hybridization signal located in a specific region of the chromosome. ND = not determined.

Species	Male Diploid Chromosome Number (2n)	Location of Autosomal C-Heterochromatin	(GATA)_4_	(AG)_10_	(AC)_10_	(AAG)_5_
Tribe Triatomini	South Lineage					
*Triatoma infestans* (Andean group)	2n = 20A + XY	7–9 II in 1 or 2 ends [36]	A = 7–9 II H Y = yes X = block	A = 7–9 II H Y = yes X = no	A = 10 II Eu Y = no X = yes	A = 10 II Eu Y = no X = yes
*T. infestans* (non-Andean group)	2n = 20A + XY	2–4 II in 1 or 2 ends [36]	A = 2–4 II H Y = yes X = no	A = 2–4 II H Y = yes X = no	A = 10 II Eu Y = no X = yes	A = 10 II Eu Y = no X = yes
*T. delpontei*	2n = 20A + XY	9–10 II in only one end [37]	A = 9–10 II H Y = yes X = block	A = 9–10 II H Y = block X = no	A = 10 II Eu Y = no X = block	A = 10 II Eu + 1 II dot Y = no X = no
*T. patagonica*	2n = 20A + XY	10 II in 1 or 2 ends [40]	A = 10 II H Y = yes X = block	A = 8–10 II H Y = yes X = no	A = 10 II Eu Y = no X = yes	A = 10 II Eu Y = no X = yes
*T. sordida*	2n = 20A + XY	6–8 II in 1 or 2 ends [41]	A = no Y = yes X = no	A = no Y = yes X = no	A = 10 II Eu Y = no X = yes	A = 10 II Eu Y = no X = yes
Tribe Triatomini	Dispar Lineage					
*Triatoma boliviana*	2n = 20A + XY	2–3 II with C-dots [31]	A = 2–3 II dots Y = yes X = no	A = no Y = yes X = no	A = 10 II Eu Y = no X = yes	ND
*T. carrioni*	2n = 20A + XY	2–3 II with C-dots [31]	A = 2–3 II dots Y = yes X = no	A = no Y = yes X = no	A = 10 II Eu Y = no X = yes	ND
Tribe Triatomini	North Lineage					
*Triatoma barberi*	2n = 20A + X_1_X_2_Y	10 II in both ends [31]	A = no Y = yes X_1_= no X_2_= yes	A = no Y = yes X_1_= no X_2_= yes	A = 10 II Eu Y = no X_1_= yes X_2_= no	A = 10 II Eu Y = no X_1_= yes X_2_= no
*T. dimidiata*	2n = 20A + X_1_X_2_Y	10 II with C-dots in both ends [37]	A = no Y = yes X_1_/X_2_ = no	A = no Y = yes X_1_/X_2_ = no	A = 10 II Eu Y = no X_1_/X_2_ = yes	A = 10 II Eu Y = no X_1_/X_2_ = yes
*T. gerstaeckeri*	2n = 20A + X_1_X_2_Y	Without C-bands [29]	A = no Y = yes X_1_/X_2_ = no	A = no Y = yes X_1_/X_2_= no	ND	ND
*T. nitida*	2n = 20A + X_1_X_2_Y	2 II almost entirely C-heterochromatic [31]	A = 1 II Eu Y = yes X_1_/X_2_ = no	A = no Y = yes X_1_/X_2_ = no	A = 9 II Eu Y = no X_1_= no X_2_ = yes	A = 10 II Eu + 1 II dot Y = no X_1_/X_2_ = yes
*T.* *protracta*	2n = 20A + X_1_X_2_Y	10 II in 1 or 2 ends [37]	A = no Y = yes X_1_/X_2_= no	A = no Y = yes X_1_/X_2_ = no	A = 10 II Y = no X_1_/X_2_ = yes	A = 10 II Eu Y = no X_1_/X_2_ = yes
*T. recurva*	2n = 20A + X_1_X_2_Y	Without C-bands [29]	A = no Y = yes X_1_/X_2_ = no	A = 1 II dot Y = yes X_1_/X_2_ = no	ND	ND
*T. rubida*	2n = 20A + X_1_X_2_Y	Without C-bands [29]	A = no Y = yes X_1_/X_2_ = no	A = no Y = yes X_1_/X_2_ = no	ND	ND
*T. rubrofasciata*	2n = 22A + X_1_X_2_Y	11 II in both ends [29]	A = no Y = yes X_1_/X_2_ = no	A = no Y = yes X_1_/X_2_ = no	A = 11 Eu Y = no X_1_/X_2_ = yes	A = 11 II Eu Y = no X_1_/X_2_ = yes
*T. sanguisuga*	2n = 20A + X_1_X_2_Y	10 II in 1 or 2 ends [29]	A = no Y = yes X_1_/X_2_ = no	A = 1 II dot Y = yes X_1_/X_2_ = no	ND	A = 10 II Eu Y = no X_1_/X_2_ = yes
*Paratriatoma lecticularia*	2n = 20A + XY	10 II in both ends [31]	A = no Y = yes X = no	A = no Y = yes X = no	A = 10 II Eu Y = no X = yes	A = 10 II Eu Y = no X = yes
*Mepraia spinolai*	2n = 20A + X_1_X_2_Y	10 II in 1 or 2 ends [29]	A = 10 II H Y = yes X_1_ = block X_2_ = no	A = no Y = yes X_1_/X_2_ = no	A = 10 II Eu Y = no X_1_/X_2_ = yes	A = 10 II Eu + 1 II dot Y = no X_1_/X_2_= yes
*Panstrongylus chinai*	2n = 20A + X_1_X_2_Y	10 II in 1 or 2 ends [31]	A = no Y = yes X_1_/X_2_ = no	A = no Y = yes X_1_/X_2_ = no	A = 10 II Eu Y = no X_1_/X_2_ = yes	A = 10 II Eu Y = no X_1_/X_2_ = yes
*P. geniculatus*	2n = 20A + X_1_X_2_Y	4–6 II in 1 or 2 ends with C-blocks [31]	A = 4–8 II Y = yes X_1_/X_2_ = no	A = no Y = yes X_1_/X_2_ = no	A = 10 II Eu Y = no X_1_/X_2_ = no	A = 10 II Eu Y = no X_1_/X_2_ = dots
*P. lutzi*	2n = 20A + X_1_X_2_X_3_Y	Without C-bands [29]	A = no Y = yes X_1_X_2_X_3_ = no	A = no Y = yes X_1_X_2_X_3_ = no	A = 10 II Eu Y = no X_1_X_2_X_3_ =yes	A = 10 II Eu Y = no X_1_X_2_X_3_ = yes
*P. megistus*	2n = 18A + X_1_X_2_Y	Without C-bands [31]	A = no Y = yes X_1_/X_2_ = no	A = no Y = yes X_1_/X_2_ = no	A = 9 II Eu + 1 II dot Y = no X_1_/X_2_ = yes	A = 9 II Eu Y = no X_1_/X_2_ = yes
*P. noireaui*	2n = 20A + XY	8–10 II in 1 or 2 ends [42]	A = no Y = yes X = no	A = no Y = yes X = no	A = 10 II Eu Y = no X = yes	A = 10 II Eu Y = no X = yes
*P. rufotuberculatus*	2n = 20A + X_1_X_2_Y	8–10 II in 1 or 2 ends [31]	A = no Y = yes X_1_/X_2_ = no	A = no Y = yes X_1_/X_2_ = no	A = 10 II Eu Y = no X_1_/X_2_ = yes	A = 10 II Eu Y = no X_1_/X_2_ = yes
Tribe Rhodniini						
*Rhodnius prolixus*	2n = 20A + XY	Without C-bands [31]	Without signals	Without signals	Without signals	Without signals
*R. ecuadoriensis*	2n = 20A + XY	Without C-bands [31]	Without signals	Without signals	Without signals	Without signals

## Data Availability

The data presented in the study are available in the article.

## Supplementary Materials

The following supporting information can be downloaded at: https://www.mdpi.com/article/10.3390/insects14090772/s1, Table S1: Geographic origin of the individuals studied.
