# Supplementary material for: Differential Spreading of Microsatellites in Holocentric Chromosomes of Chagas Disease Vectors: Genomic and Evolutionary Implications
Source: Insects. 2023 Sep 19;14(9):772. doi: 10.3390/insects14090772 (PMC10531928; doi:10.3390/insects14090772)
Supplement: Supplementary file 1 [file insects-14-00772-s001.zip › insects-2538626-supplementary.pdf]

insects

Supplementary Materials

Differential Spreading of Microsatellites in Holocentric Chromosomes of Chagas Disease Vectors: Genomic and Evolutionary Implications

Table S1. Geographic origin of the individuals studied.

	Species	Geographic origin
**Tribe Triatomini**	**South American lineage**	
infestans complex	*Triatoma infestans.* (Andean group)	Bolivia, Potosí, Palquiza, Sylvatic. 21º31'41''S, 65º45'04''W<br>Bolivia, La Paz, Loayza, Sapini, Peridomestic. 16º48'55''S, 67º42'21''W
	Triatoma infestans (non-Andean group)	Argentina, Chaco, Tres Estacas, Per. 26º54'30''S, 51º40'23''W<br>Argentina, Santiago del Estero, Silipica, Per. 28º13'25''S, 64º07'13''W
	Triatoma delpontei	Bolivia, Santa Cruz, Tita, S. 18º34'31''S, 62º40'05''W
rubrovaria complex	*Triatoma patagonica*	Argentina, Santiago del Estero, Mitre, Per. 29º24'50''S, 62º47'23''W
sordida complex	*Triatoma sordida*	Brazil, Minas Gerais, Montes Claros, Per. **16º44'6''S, 43º51'42''W**
	**Dispar lineage**	
	Triatoma boliviana	Bolivia, La Paz, Muñecas, Chuma, Per. 15º28′51″S, 68º53′54″W
	Triatoma carrioni	Perú, Piura, Ayabaca, Socchabamba, S. 4º35′43″S, 79º40′09″W
	**North American lineage**	
protracta complex	*Triatoma barberi*	Mexico, Querétaro, La Cueva. Per. 20º29'04''N, 100º26'20''W
	Triatoma protracta	USA, Arizona. Insectary Justin Schmidt.
dimidiata complex	*Triatoma dimidiata*	Guatemala, Baja Verapaz, different localities. Domestic.
nitida complex	*Triatoma nitida*	Guatemala, Quiché, Zacualpa, D. 15º01'34''N, 90º52'42''W
	Triatoma rubida	USA, Arizona, Tucson, S.
phyllosoma complex	*Triatoma recurva*	USA, Arizona, Tucson, S.
	Triatoma gerstaeckeri	USA, Texas, Frio Cave, S.
rubrofasciata complex	*Triatoma rubrofasciata*	Vietnam, Hanoi, Tu Liem district. Per. 21º02'48''N, 105º44'54''E
(uncertain complex)	*Triatoma sanguisuga*	USA, Louisiana, S.
lecticularia complex	*Paratriatoma lecticularia*	USA, Texas, S.
spinolai complex	*Mepraia spinolai*	Chile, Atacama, Chañaral, Inca Oro, D. 26º45'20''S, 69º54'16''W

	Panstrongylus chinai	Perú, Lambayeque, Chiclayo, Zaña, D. 6º54'15''S, 79°34'27''W
	Panstrongylus geniculatus	Colombia, Santander, Bucaramanga, S. 7º07'31''N, 73º7'11''W
	Panstrongylus lutzi	Brazil, Ceará, Várzea Alegre, Per. 06º47'20''S, 39º17'00''W
	Panstrongylus megistus	Brazil, Rio de Janeiro, Botanical Garden, S.
	Panstrongylus noireaui	Bolivia, La Paz, Muñecas, Camata, Per. 15º14'22''S, 68º44'52''W
	Panstrongylus rufotuberculatus	Colombia, La Guajira, Gumake, Per. 111º12'15''N, 73º14'58''W<br>Colombia, Antioquia, Amalfi, Per. 6º54′33″N, 75º04′36″W
**Tribe Rhodniini**		
	**Prolixus lineage**	
	Rhodnius prolixus	Colombia, S. Insectary CDC, Atlanta, USA.
	**Pallescens lineage**	
	Rhodnius ecuadoriensis	Perú, La Libertad, Gran Chimú, Simbrón, D. 7º33'13''S, 78º33'26''W
